# Longitudinal micro-endoscopic monitoring of high-success intramucosal xenografts for mouse models of colorectal cancer

**DOI:** 10.7150/ijms.35666

**Published:** 2019-09-20

**Authors:** Bjorn Paulson, Ick Hee Kim, Jung-Man Namgoong, Young Gyu Kim, Sanghwa Lee, Youngjin Moon, Dong-Myung Shin, Myung-Soo Choo, Jun Ki Kim

**Affiliations:** 1 Biomedical Engineering Research Center, Asan Institute for Life Sciences, Asan Medical Center, 88, Olympic-ro 43-gil, Songpa-gu, Seoul 05055, Republic of Korea; 2 Wake Forest Institute for Regenerative Medicine, Wake Forest School of Medicine, 391 Technology Way, Winston-Salem, NC 27101, USA; 3 Department of Surgery, Asan Medical Center, University of Ulsan College of Medicine, 88, Olympic-ro 43-gil, Songpa-gu, Seoul 05055, Republic of Korea; 4 Department of Convergence Medicine, University of Ulsan College of Medicine, 88, Olympic-ro 43-gil, Songpa-gu, Seoul 05055, Republic of Korea; 5 Department of Biomedical Sciences, Asan Medical Center, University of Ulsan College of Medicine, 88, Olympic-ro 43-gil, Songpa-gu, Seoul 05055, Republic of Korea; 6 Department of Physiology, Asan Medical Center, University of Ulsan College of Medicine, 88, Olympic-ro 43-gil, Songpa-gu, Seoul 05055, Republic of Korea; 7 Department of Urology, Asan Medical Center, University of Ulsan College of Medicine, 88, Olympic-ro 43-gil, Songpa-gu, Seoul 05055, Republic of Korea

**Keywords:** Orthotopic injection, Colorectal cancer, mouse models, microendoscopy, side-view endoscopy, fluorescence imaging

## Abstract

Colorectal cancer (CRC) is one of the most frequently lethal forms of cancer. Intramucosal injection allows development of better mouse models of CRC, as orthotopic xenografts allow development of adenocarcinoma in the submucosa of the mouse colon wall. In this paper, a method of orthotopic injection is monitored longitudinally using cellular-resolution real-time *in vivo* fluorescence microendoscopy, following the injection of three different cell lines: 3T3-GFP to confirm immunosuppression and HCT116-RFP cells to model CRC. Adenoma formation is first observable after 7 to 10 days, and by use of 33 G needles a tumor induction rate of greater than 85% is documented. An additional experiment on the injection of rapamycin reveals drug efficacy and localization between 24 and 48 hours, and suggests the promise of real-time cellular-resolution fluorescence micro-endoscopy for developing longitudinal therapy regimes in mural models of CRC.

## Introduction

Colorectal cancers (CRC) have the second highest mortality rate among cancers worldwide, accounting for around 11% of cancer deaths in men and 7% in women, and exhibit the highest mortality rate among intestinal diseases 1. With the goal of developing novel cancer therapeutics, several standard methods have been developed for the precipitation of colorectal cancer (CRC) in mouse models: genetically engineered mice which recapitulate known cancerogenic genotypes 2, chemically induced but non-specific tumorigenesis 3, xenografts of human tumor tissue into immune-compromised mice 4, and xenografts of human cancer tissue into mice with humanized immune systems 5. While mural tumors in genetically engineered mouse models give realistic tumor expression in a realistic microenvironment, accurately demonstrating the pathology and mechanisms of several tumor types, their performance for the discovery of molecularly-targeted treatment compounds has been limited. In contrast, the efficacy of compounds on human tumor-derived cell lines and transplanted cancer tissue in the mural model has been predictive of phase 2 clinical trial performance 6. In addition, transplantation of tumor tissue allows for fine control of tumor location in comparison to spontaneously tumorigenic transgenic mouse models 7. As a result, xenografts of cancerous cells and tissues are the primary method for inducing colorectal cancer in mouse models.

Typical procedure for the xenograft of colorectal tissue into mouse models is to section fresh surgical tissue into small pieces, followed by implantation either subcutaneously by injection, or orthotopically via surgery. The success or failure of engraftment can be ascertained after four to six months, and immunocompromised mouse models have resulted in tumor induction rates of up to 95%, although most investigators report take rates of about 75% 6,8. Surgical implantation has several limitations. Implantation requires significant surgical time, feedback on procedure success is slow, failure rates are high, and transplantation precisely to the colon epithelium is difficult. In order to overcome these difficulties, several groups have recently developed less invasive and more precise methods for orthotopic xenografts of CRC tissues, including enema-based acid treatment 9, a specially-designed sponge 10, and intramucosal (orthotopic) injection 4,11-14.

Where the development of minimally invasive methods for orthotopic transplantation has enabled precise and consistent tumor placement, the development of micro-endoscopy has recently enabled *in situ* targeting and evaluation of CRC models. Optical measurement using a micro-endoscope gives real-time data about the surface optical properties of organs in the living organism 15*.*

Using a working channel on a laparoscope, our group has demonstrated ovarian injection of chemo-resistant cell cultures 16, while Roper *et al.* have successfully used colonoscopy to target individual submucosal tumors at 0.5 cm intervals along the mural colon 11. By avoiding the use of surgery and reaching the target microenvironment directly, injection-based orthotopic xenografts promise a more realistic microenvironment, and the narrow diameter of the needle minimizes the chance of pneumoperitoneum even in the case of colon perforation, while increasing tumor induction and survival rates. Thereby researcher time, money, and animals may be saved 14.

In this study, a modified method for the orthotopic transplantation of colorectal cancer (CRC) xenographs is presented and studied. In contrast to previous injection methods 11-14, the method presented does not require the use of bespoke needles, yet still achieves CRC adenoma formation in more than 85% of mouse models. Longitudinal, real-time monitoring following injection is performed both via commercially available colonosope, as in previous work, and via a custom side-view laser-scanning confocal micro-endoscope, which allows cellular-resolution imaging of the colon lumen. Tumor formation is confirmed by confocal microscopy and histology. Taken together, these results demonstrate the first in vivo cellular-resolution longitudinal monitoring of orthotopically injected CRC models, to the best knowledge of the authors. Finally, feasibility of the injection procedure for localized treatment and chemically-enhanced optical biopsy is assessed by injection of rapamycin into LC3-GFP+ mice, and found promising for the localized assessment of future cancer therapies.

## Materials and Methods

### Cell lines and preparation

Human colorectal cancer cell line HCT116 was prepared at the Wellman Center for Photomedicine, Massachusetts General Hospital (MGH), while 3T3-GFP-expressing fibroblast cells were graciously donated by the Center for Computational and Integrative Biology (CCIB), Massachusetts General Hospital (MGH). A red fluorescent protein (RFP) lentivirus (Lenti-Red, Biogenova, Rockville, USA) was transfected to induce stable RFP expression in HCT116. Cells were cultured in RPMI1640 medium supplemented with 10% fetal bovine serum, 100 U/ml penicillin, 0.1 mg/ml streptomycin and 2 mM L-glutamine. The medium was changed every 1-2 days. For each injection, 3.0 × 10^5^ cells were administered at a density of 10^4^ cell µL^-1^.

### Rapamycin and fluorescent microparticles

Rapamycin and fluorescent micro particles (Sigma-Aldrich) were prepared for injection. Fluorescent particles were excited at 636 nm for fluorescence at 686 nm. Rapamycin was dissolved in DMSO (dimethyl sulfoxide), then added in 1:24 proportion to a solution of 10% polyethylene glycol (MW avg. = 400 Da).

### Mouse preparation

Female BALB/c nude mice and LC3-GFP mice (Jackson Laboratories), seven to sixteen weeks old, were raised in a specific pathogen free environment, controlling for pathogens, temperature, humidity, atmospheric contaminants, lighting, and sound. Nude mice were split into three groups of 7 mice each, and LC3-GFP mice were split into two groups: a LC3-GFP+ control group, subject to a sham procedure with injection of fluorescent beads; an LC3-GFP+ treatment group subject to injection of fluorescent beads and rapamycin. One nude mouse group each was subject to injection of 3T3 cells, and HCT116-RFP cells. All animal studies were conducted in accordance with the policies of the NIH Guide for the Care and Use of Laboratory Animals and approved by the Institutional Animal Care and Use Committee (IACUC) of Massachusetts General Hospital (MGH2007N0000110).

### Intramucosal xenografts and forward-view colonoscopy

**Fig 1:**
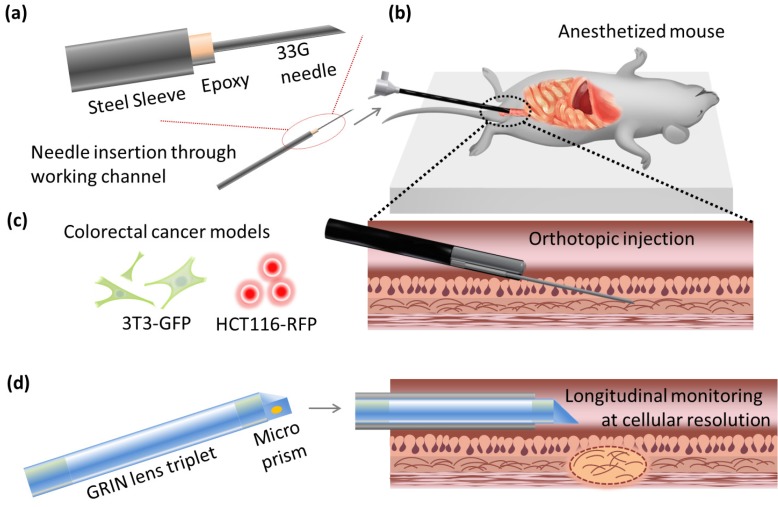
** Schematic of the experiments.** (a) Minimally invasive transplantation needle designed from commonly available parts. (b) Needle is inserted through the working channel of a forward-viewing colonoscope for (c) orthotopic injection of CRC cells into mouse models. (d) Side-view GRIN lens triplet micro-endoscope probes are applied for longitudinal monitoring of neoplasm at cellular resolution *in vivo*.

Before xenografts were performed, an extended needle was prepared for insertion through the endoscope's working channel. A 33G needle was bonded to a 2 Fr diameter hollow stainless steel sheath using a thermal-curing epoxy (Thorlabs) (**Fig 1(a)**). The connection to the sheath was then wrapped with parafilm (Bemis, WI, USA) to protect the epoxy from mechanical strain during insertion into the curved 3 Fr diameter working channel of the colonoscope.

For all injections, mice were anesthetized intraperitoneally with 75 mg kg^-1^ ketamine and 15 mg kg^-1^ xylazine, according to standard protocols, and placed on a heated pad for the maintenance of homeostasis. LC3-GFP mice were shaved using clippers and depilatory cream. The colon was washed with 0.5 mL phosphate buffered saline (PBS) at 37 degrees centigrade, using a rubber-tipped syringe. A three-axis micro-stage was used to direct and guide insertion of a 2.8 mm diameter Coloview® miniature colonoscope (Karl Storz) 2 cm proximally from the end of the colon, while a 33G needle was staged near the end of the working channel (**Fig 1(b)**). The needle was then extended into the colon tissue, and the desired cell solution was injected (**Fig 1(c))**. All cells were administered in batches of 3.0 × 10^5^ cells at a density of 10^4^ cell µL^-1^. Fluorescent microbeads in buffered saline solution and rapamycin solutions were prepared and injected to a total volume of 100 μL.

### Side-view confocal micro-endoscopy and fluorescent cellular imaging

Cellular resolution images were captured *in vivo* with a custom built side-view confocal micro-endoscope (**Fig 1(d))**. A side-view gradient-index (GRIN) relay micro-endoscopy probe of length 5.5 cm and diameter 1.2 mm was assembled using three GRIN lenses and an angled prism, cementing lenses together as previously described 17. The side-view probe has a field of view of 220 microns, and achieves transverse and lateral resolutions of 1 μm and 11 μm in air, respectively, which is sufficient for image capture at single-cell resolution. It was connected to a custom built laser scanning confocal microscope via a 4*f* lens relay 18.

A three-axis micro-stage was used to guide the endoscope along the colon, and allowed measurement of probe position relative to the distal end of the colon. Green fluorescent protein (GFP) and red fluorescent protein (RFP) were excited using continuous wave 488 nm and 532 nm laser diodes, respectively, which were scanned over the field of view at 30 fps via resonant scanning mirrors. Photomultiplicative detectors (PMT) were used in tandem with dichroic filters to capture emitted fluorescence while avoiding noise caused by the excitation laser and autofluorescence. Green fluorescent protein has a fluorescence peak at 525 nm, while RFP emits at 607 ± 15 nm, respectively. Fluorescent microbeads were excited using a 636 nm laser and emissions were detected at 680 ± 21 nm.

Large-field-of-view images were generated by compositing smaller endoscopic images into a mosaic as previously described 18. Average fluorescence intensity was quantified by separating out the color channel for the relevant fluorescence signal and taking the average value of that channel for all nonzero pixels in the image. LC3-GFP cells undergoing autophagy were counted by thresholding the GFP channel at 60% of its saturation value and counting the number of clumps of nine or more connected pixels above the threshold value. The autophagy cellular density was then calculated by dividing by the field of view.

### Histology

After terminating longitudinal observations, the distal colons were harvested and tumors were removed from the colon after sacrifice. Tumor diameters were measured with digital calipers before tumor sections were cut, fixed in formalin, and stained under hematoxylin and eosin dyes for micrographs. Neoplasms were verified to be of the injected fluorescent cell lines by *ex vivo* confocal fluorescence microscopy. Histology results were matched with forward colonoscopy images to calibrate tumor size measurements for longitudinal *in vivo* tumor size estimation.

## Results and Discussion

The demonstration of consistent orthotopic tumorigenesis without immune rejection is necessary prior to the longitudinal study of orthotopic mural models of CRC. Previously, Zigmond *et al.* have reported, using 30G needles, that orthotopic transplantation of injected cell counts of 10^5^ or higher result in increased colonic obstruction and higher-grade tumors over a fixed period of 3 weeks 12 compared to smaller cell counts. However, Roper *et al* demonstrated the use of a smaller needle gauge to be well suited to infusing CRC-inducing transgenic compounds in the lamina propria of a mouse model due to its reduced risk of complications from colon perforation 11. In this work, a standard 33G hypodermic needle with a 30 degree bevel was applied, necessitating confirmation of tumor formation efficacy at the desired cell counts.

**Fig 2:**
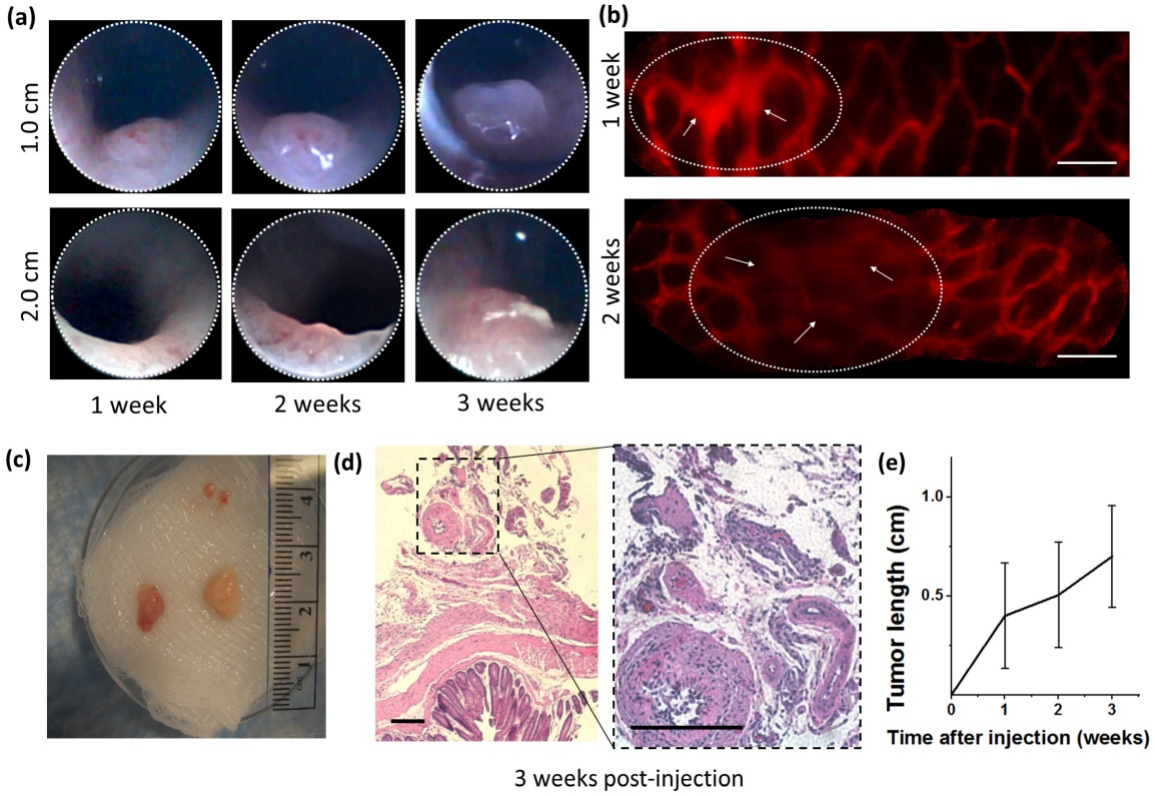
** Orthotopic injection and tumorigenesis from 3T3-GFP cells in BALB/c nude mice.** (a) Longitudinal forward-view colonoscope observation following injection shows tumor formation over a period of 3 weeks. (b) In an induced tumor 1.0 cm from the distal end of the colon, patterns of microvascular deformation (white arrows) were observed by side-view endoscopy after 1 and 2 weeks. Scale bars 100 µm. (c) Size comparison of the harvested tumors. (d) Micrograph of excised tumor tissue following hematoxylin and eosin stain. Scale bars 250 µm. (e) A plot of the tumor size progression observed longitudinally by front-view colonoscopy and combined with post-sacrifice direct size measurements. All results are indicative of successful transplantation without immune rejection.

To verify the efficacy of tumor generation for our modified injection process, GFP-expressing mouse embryonic fibroblast 3T3 cells (3T3-GFP), which are commonly used for xenograft studies of immune rejection, were injected into BALB/c nude mice. At distances of 1.0 and 2.0 cm from the distal end of the colon, the injection locations on the mucosa were imaged weekly over a period of 3 weeks by colonoscopy using the Coloview® endoscope and by side-view micro-endoscopes. Representative longitudinal tumor growth is shown in **Fig 2(a)**. Distinct neoplasia was evident after a two-week period, and adenoma formation was clearly observed after three weeks. For the first two weeks after orthotopic implantation, mouse colon vasculature was monitored using the side-view fluorescent confocal micro-endoscope after rhodamine dextran intravascular (IV) injection. While bulging was observed due to formation of tumor in the colon epithelium immediately after injection, the deformed microvascular patterns were observed in patches over the bulged area as shown in **Fig 2(b)**. As the week progressed, the area affected by this bulging pattern appeared to become wider.

After 3 weeks, mice were sacrificed and colons were harvested for histology as shown in **Fig 2(c,d)**. Overall, tumor growth was successful and tumor size ranged between 3 mm (6 mm^3^) and 8 mm (64 mm^3^). Histology showed deformities in the submucosa and muscularis consistent with neoplasm. All samples were injected submucosally, and no deaths or colon perforations were observed. The tumors were successfully generated at a tumor induction rate of 85% (N=14 in 7 mice), where the tumor induction rate is defined as the percent of orthotopic injections which resulted in a tumor within a 14 day period.

**Fig 3:**
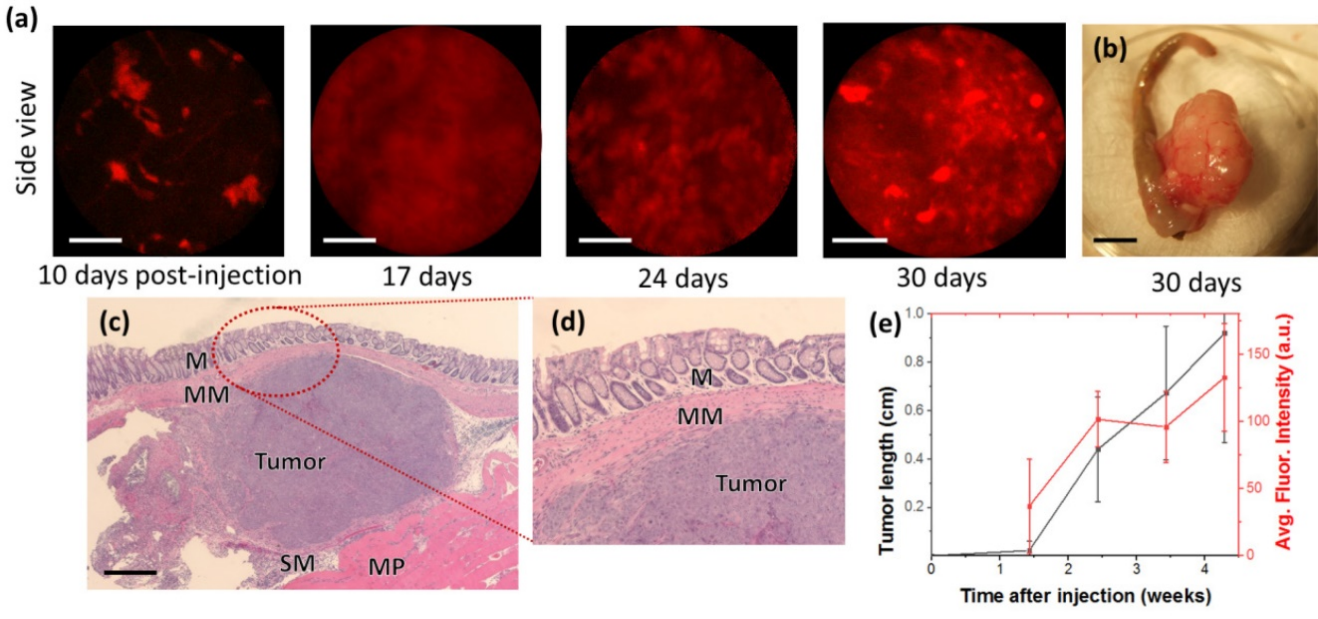
** Cellular resolution real-time longitudinal monitoring of HCT116-RFP cells after orthotopic injection.** (a) Side-view cellular-resolution micro-endoscopy post-injection shows visible neoplasm after 10 days. (b) Gross image of excised tumor tissue. (c) Hematoxylin-and-eosin-stained histology of colon section showed tumor formation in the tissue layers of the colon; M:Musosa, MM:Muscularis mucosae, SM:Submucosa, MP:Muscularis Propria. (d) Higher-magnification images of the circled region (d). Scale bars: (a) 50 µm, (b) 5 mm, (c) 500 µm.

While 3T3-GFP cells demonstrate immune suppression, the study of human cancer cells in mouse models of CRC is more directly demonstrated by the HCT116-RFP cell line, a human CRC line expressing red fluorescent protein (RFP), which is of interest due to its visibility in side-view fluorescence confocal endomicroscopy. Identical quantities (3 × 10^5^ cells) of HCT116-RFP were delivered submucosally into BALB/c nude mice, and examined by fluorescent side-view endoscopes 10, 17, 24, and 30 days after implantation, as shown in **Fig 3(a)**.

Using cellular-resolution side-view colonoscopy, injected HCT116-RFP cells could be observed at 10 days. After 24 days, imaging was complicated by restriction of the colon around the tumor sites.

While neoplasm was not distinctly evident 10 days after injection in the front-view colonoscope, cellular-level development of neoplasm was evident in the side-view images from the same time period. It should be noted that the expression of fluorescent protein can be induced in cell and tissue cultures virally, and thus the monitoring of injection sites by side-view micro-endoscopy presents a method for the early detection of CRC model mice for which tumorigenesis is not successful due to immune rejection or other factors. This potentially decreases the time required for orthotopic CRC transplantation experiments by up to a week. We could confirm that the growth speed of the tumor is dependent of species and aggressiveness of the cell lines. This was also apparent by inspection of colons *ex vivo,* as large growths were visible external to the colon, as shown in **Fig 3(c)**. The HCT116-RFP tumors were also imaged by histology after one month. As shown in **Fig 3(c)** and **Fig 3(d)**, the growth of tumors was observed between the muscularis mucosae and muscularis propria.

While several authors have demonstrated the orthotopic transplantation of genetically modified cells and tissues 12,14, understanding of the localization properties and cellular behavior of genetically inert pharmaceuticals in the *in vivo* environment is also desirable. To this end, rapamycin was injected orthotopically into LC3-GFP expressing transgenic mice and monitored longitudinally at cellular resolution by side-view micro-endoscopy.

Mice expressing green fluorescent LC3 protein (LC3-GFP) allow for the highly specific visualization of autophagy *in vivo*, as the LC3 membrane protein binds to autolysosome membranes and is degraded during autophagy, expressing punctate fluorescence signals during autophagic processes as a result 19,20. Autophagy is chiefly regulated by the mammalian target of rapamycin (mTOR), although mTOR-independent autophagy pathways also exist 21. Thus, in LC3-GFP expressing transgenic mice, areas of high rapamycin concentration may be expected to show cells with significantly increased fluorescence spots, as negative regulation of autophagy is inhibited. While the sub-cellular-sized puncta are not visible in standard colonoscopy, individual cells with abnormally increased fluorescence are distinguishable at the resolution of the side-view GRIN micro-endoscope probe.

**Fig 4:**
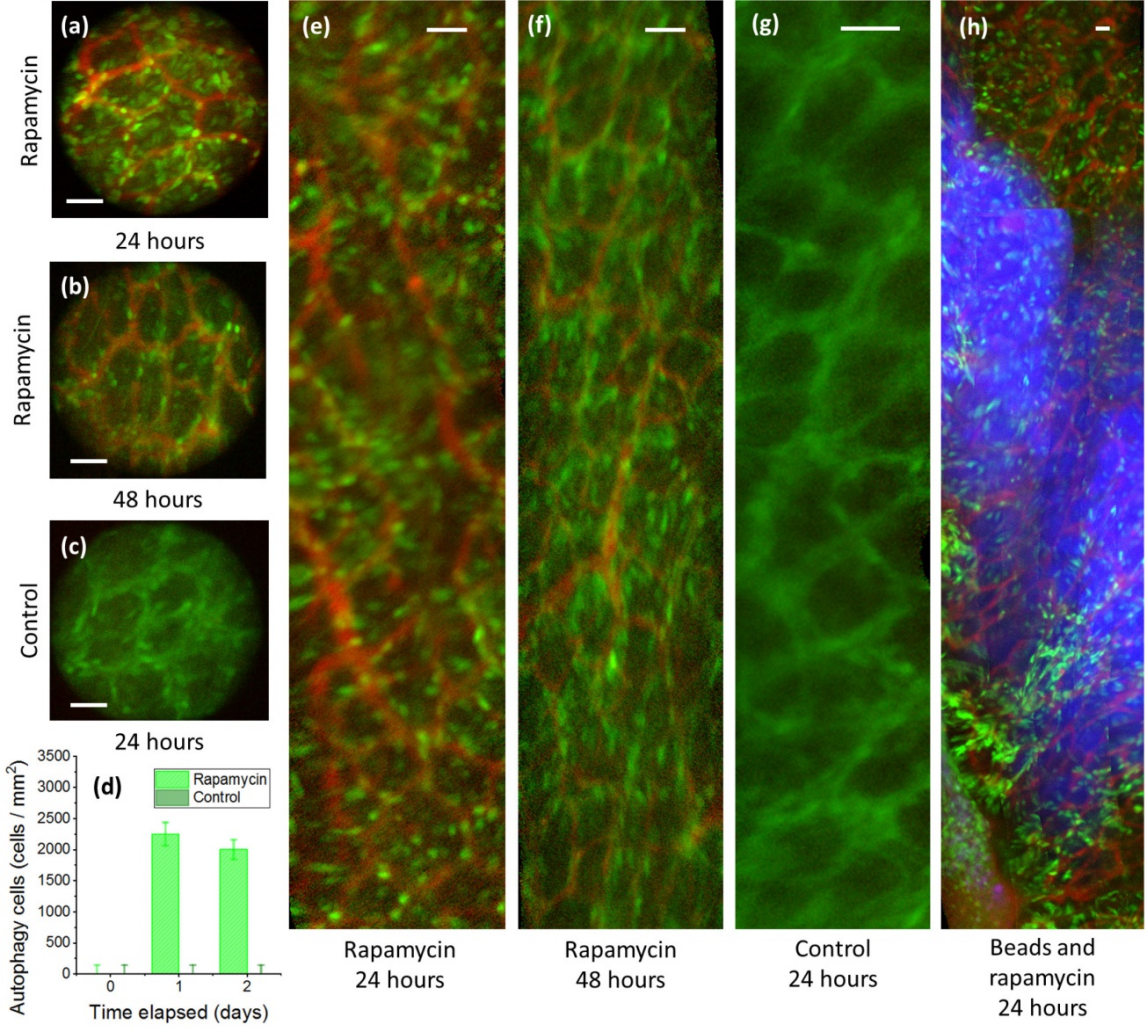
** Cellular resolution side-view microendoscope images following orthotopic injection of rapamycin in the mural colon.** (a,c,d,e) Rapamycin injection sites 24 hours post-injection show higher fluorescence than (b,f) after 48 hours. (c,f) Control image without Rapamycin injection. (d) Rapamycin treated mice show higher autophagy than controls for all time periods. (h) Rapamycin shows slightly increased diffusion compared to concurrently injected fluorescent microparticles after a 24 h period. Green = GFP, bright green = autophagy, red = rhodamine dextran, blue = fluorescent beads. Scale bars, 40 microns.

By delivering rapamycin to LC3-GFP mice, the *in vivo* localization and duration of rapamycin may be assessed via the fluorescence of cell autophagy, as shown in **Fig 4**. In this study, a treatment group of tumor-free LC3-GFP+ mice was orthotopically injected with a mix of relatively immobile fluorescent nanospheres and rapamycin, while a tumor-free control group was subject to the same procedure without rapamycin. Although there is a risk that punctate GFP signals may result from autofluorescence and from LC3-GFP trapped in protein aggregates 22, these may be limited to the effects of rapamycin by comparison of fluorescence relative to the control group. Fluorescent nanospheres were used to mark the injection location, allowing repeated monitoring of the induced autophagy, and thereby the persistence of rapamycin, by side-view confocal micro-endoscopy. Over a 48-hour period, strong cellular signals likely indicative of autophagy were observed in the rapamycin-treatment group, but not in the control group.

Although the field of view of the GRIN microendoscope is limited, the measuring and marking of multiple points as well as the composition of images from several passes and rotations of the side-view microendoscope allow the generation of larger, contiguous images 17,23. Several such post-processed images are used in Fig. 4(e-h) to show the extent of the spread of the rapamycin-derived autophagy.

In the first 24 hours, autophagy spread slightly beyond the injection region, as marked by fluorescent beads, to be localized in an area of 10^5^~10^6^ microns^2^ for the first 24 h. (Fig. 4h). The spread of the rapamycin may be due to DMSO-assisted perfusion, or due to diffusion through the extracellular matrix. Autophagy declined between 24 and 48 h. No mouse fatalities were observed in either the control group or the treatment group (7 mice each).

In addition to its autophagic properties, rapamycin's inhibition of mTOR interrupts the signaling cascades of *KRAS*-negative CRC tumors, and targeted injection of rapamycin may be promising for treating these carcinomas in human patients 7. However, targeted intramucosal injection paired with longitudinal monitoring is also powerful tool for the development and testing of other therapeutics targeted at pre-metastatic CRCs.

## Conclusion

Through the process of orthotopic injection and transplantation of CRC cell cultures and organoids in mouse models, mural models for colorectal cancers have recently been developed with high tumor induction rates and improved accuracy to human CRC 14. This work builds on those models, demonstrating the use of side-view confocal micro-endoscopy for the minimally invasive, longitudinal, and cellular-resolution monitoring of tumor development. The submucosal injection of cell cultures was performed using standard gauge hypodermic needles, and monitored longitudinally. Tumorigenesis is demonstrated with 3T3-GFP and HCT116-RFP cells in mural models, and real-time side-view endoscopy is used to demonstrate early identification of neoplasm. Side-view endomicroscopy also allows for the observation of cellular-level features, such as the fluorescence of LC3-GFP due to autophagy, as demonstrated by the injection of rapamycin. With rapamycin, the potential for targeted orthotopic injection of pharmaceuticals is demonstrated and an alternative method for evaluation of their efficacy *in vivo* is shown, which has potential for application in future mural models of CRC.

## Abbreviations

CRC: Colorectal cancers, GFP, green fluorescent protein, RFP, red fluorescent protein, DMSO dimethyl sulfoxide
MW: molecular weight
PMT: photomultiplier tube
G: gauge
mTOR: molecular target of rapamycin
